# Supplementary material for: Marker-Independent Food Identification Enabled by Combing Machine Learning Algorithms with Comprehensive GC × GC/TOF-MS
Source: Molecules. 2022 Sep 22;27(19):6237. doi: 10.3390/molecules27196237 (PMC9572226; doi:10.3390/molecules27196237)
Supplement: Supplementary file 1 [file molecules-27-06237-s001.zip › molecules-1927591-supplementary.pdf]

Supplementary materials

Marker-Independent Food Identification Enabled by Combing Machine Learning Algorithms with Comprehensive GC × GC/TOF-MS

Bei Li [1,2], Miao Liu [3], Feng Lin [3], Cui Tai [1], Yanfei Xiong [3], Ling Ao [3], Yumin Liu [4], Zhixin Lin [1], Fei Tao [1,*] and Ping Xu [1]

Table of Contents

Table S1, S-2

Table S2, S-4

Table S3, S-4

Figure S1, S-5

Figure S2, S-6

Figure S3, S-7

[*] Corresponding author. Tel.: +86 21 34206647; fax: +86 21 34206647. *E-mail address:* taofei@sjtu.edu.cn (F. Tao)

Table S1. Samples were detected in this research.

Flavor	Identifier	Brand of Chinese liquor	Abbreviation	Origin	Serie	Brand in Chinese
Strong flavor	1	Wuliangye	WLY	Yibin Sichuan	21	五粮液
	2	Zhijiang Daqu	ZJDQ	Zhijiang Hubei	3	枝江大曲
	3	Baiyunbian	BYB	Jingzhou Hubei	3	白云边
	4	Zhongzijiu	ZZ	Fuyang Anhui	9	种子酒
	5	Shuanggou	SG	Haozhou Anhui	12	双沟
	6	Jiannanchun	JNC	Mianzhu Sichuan	12	剑南春
	7	Bandaojing	BDJ	Zibo Shandong	3	扳倒井
	8	Laijiajiangjiu	LJJJ	Renhuai Guizhou	3	赖家酱酒
	9	Shuijingfang	SJF	Ckhengdu Sichuan	3	水井坊
	10	Gujing Tribute	GJT	Haozhou Anhui	9	古井贡酒
	11	Xiaohu Tuxian	XHTX	Renhuai Guizhou	3	小糊涂仙
	12	Yanghe	YH	Suqian Jiangsu	19	洋河
	13	Dukang	DK	Luoyang Henan	6	杜康
	14	Tuopai	TP	Suining Sichuan	6	沱牌
	15	Langyatai	LYT	Qingdao Shandong	3	琅琊台
	16	Yinianchun	YNC	Tongren Guizhou	3	颐年春
	17	Yingjia Tribute	YJT	Huoshan Anhui	3	迎驾贡酒
	18	Luzhou Laojiao	LZLJ	Luzhou Sichuan	33	泸州老窖
	19	Jingzhi	JZ	Jiangzhi Shandong	6	景芝
	20	Jiuguijiu	JGJ	Jishou Hubei	3	酒鬼酒
	21	Dongjiu	DJ	Zunyi Guizhou	3	董酒

	22	Kongfujia	KFJ	Qufu Shandong	6	孔府家
	23	Maotaichun	MTC	Zunyi Guizhou	3	茅台醇
Sauce flavor	24	Maotai	MT	Zunyi Guizhou	6	茅台
	25	Jinsha	JS	Bijie Guizhou	3	金沙
	26	Guotai	GT	Renhuai Guizhou	3	国台
	27	Jiangjiu	JJ	Renhuai Guizhou	3	酱酒
Fen flavor	28	Fenjiu	FJ	Fenyang Shanxi	24	汾酒
	29	Jiangxiaobai	JXB	Chongqing	3	江小白
	30	Niulanshan	NLS	Beijing	6	牛栏山
	31	Hongxing	HX	Beijing	3	红星
	32	Jingwangwang	JWW	Beijing	3	京旺旺
	33	Laobaigan	LBG	Hengshui Hebei	6	老白干
Site flavor	34	Site	ST	Ganzhou Jingxi	6	四特
Feng flavor	35	Xifeng	XF	Baoji Shaanxi	12	西凤
Mixed flavor	36	Kouzi	KZJ	Huaibei Anhui	9	口子酒
Total	36				262	

Table S2. Plackett–Burman experimental design

Factor	Level		
	Low (−1)	Central (0)	High (+1)
A: extraction temperature (°C)	25	42.5	60
B: extraction time (min)	15	37.5	60
C: incubation time (min)	15	32.5	50
D: salt addition (M)	0	1	2
E: alcohol strength (%, *v/v*)	10	31	52

Run	Factor				
	A	B	C	D	E
1	60	60	15	2	10
2	60	15	15	0	10
3	42.5	37.5	32.5	1	31
4	60	60	50	2	52
5	25	15	50	2	10
6	42.5	37.5	32.5	1	31
7	25	60	15	0	52
8	60	15	50	0	52
9	25	60	50	0	10
10	25	15	15	2	52

Table S3. SVM model results for brands and flavors of Chinese liquors

Model	Standard	Spectral region (*m/z*)	Accuracy (%)
SVM	brands	20–400	82.56
		20–200	91.86
	flavors	20–400	95.36
		20–200	97.67

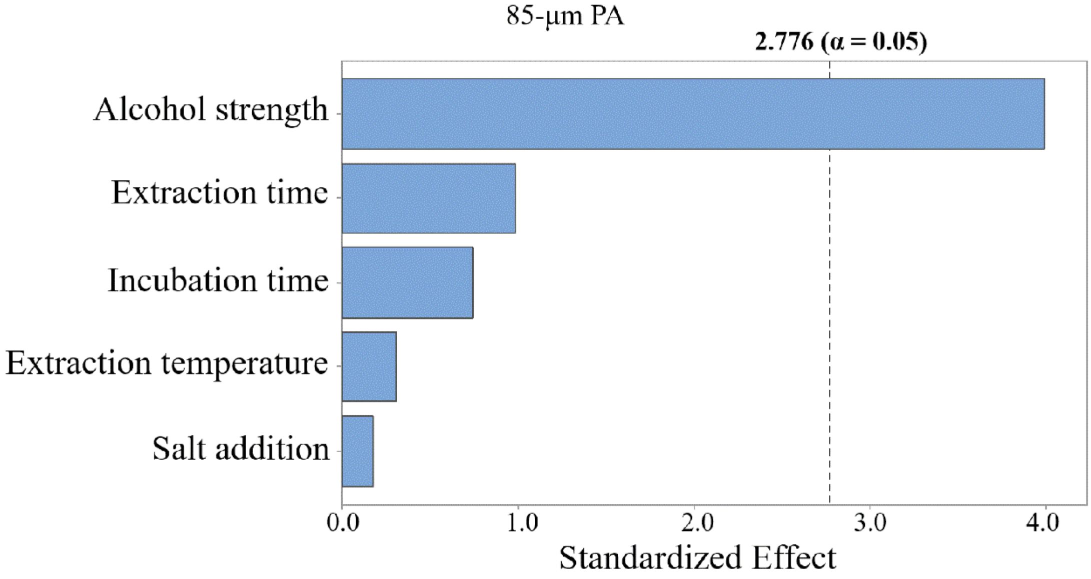

Figure S1. Standardized main effect Pareto chart, representing the estimated effects of parameters obtained from the Plackett-Burman (P-B) design for determination of 85-µm PA, Vertical line in the chart defines 95% confidence level.

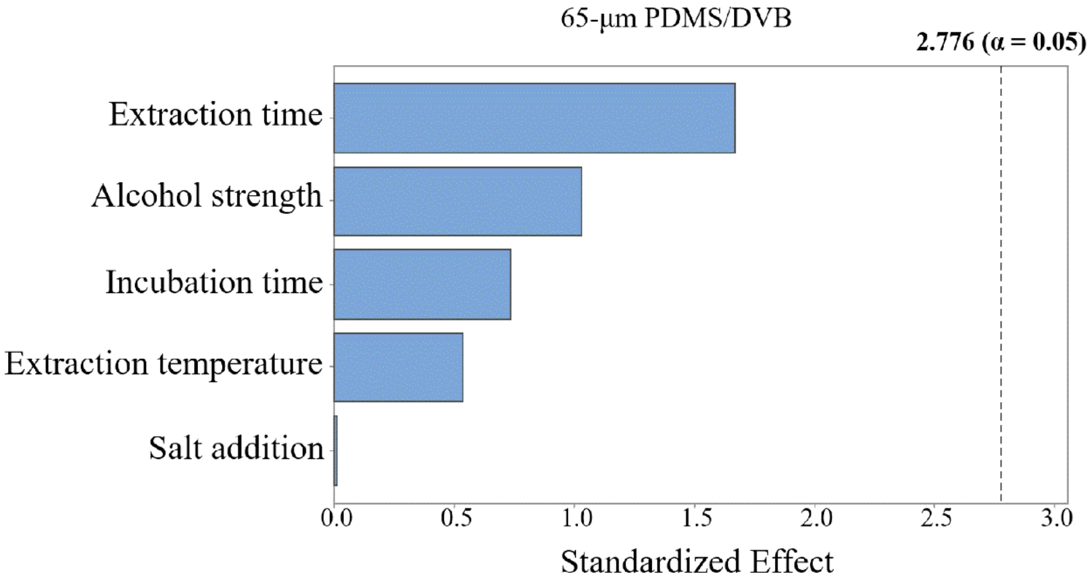

Figure S2. Standardized main effect Pareto chart, representing the estimated effects of parameters obtained from the Plackett-Burman (P-B) design for determination of 65-µm PDMS/DVB, Vertical line in the chart defines 95% confidence level.

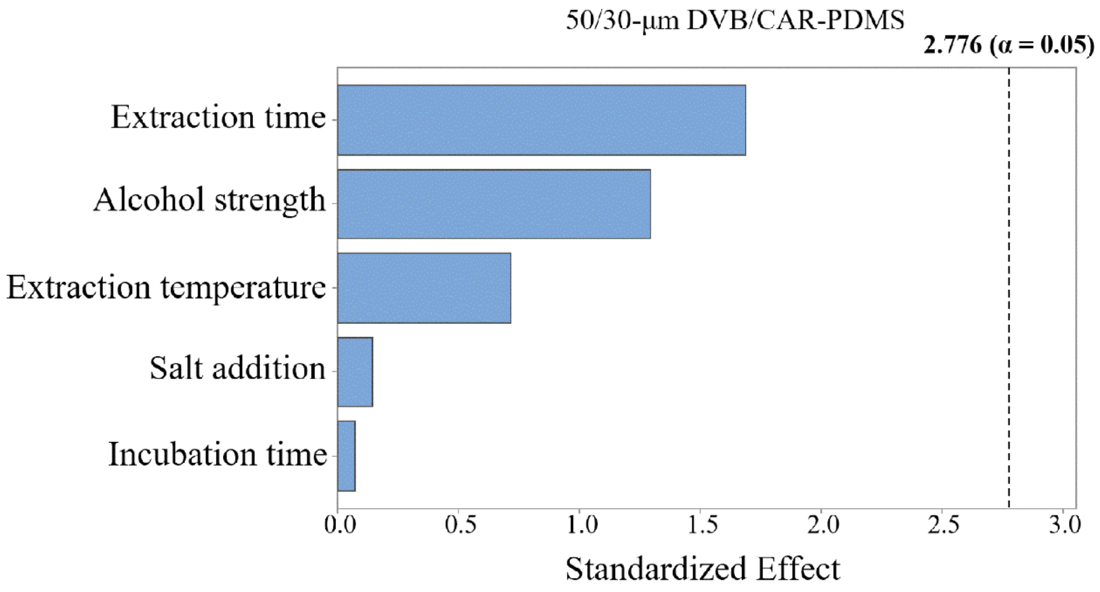

Figure S3. Standardized main effect Pareto chart, representing the estimated effects of parameters obtained from the Plackett-Burman (P-B) design for determination of 50/30-μm DVB/CAR-PDMS, Vertical line in the chart defines 95% confidence level.
